# Impact of Hepatic Encephalopathy on Clinical Characteristics and Adverse Outcomes in Prospective and Multicenter Cohorts of Patients With Acute-on-Chronic Liver Diseases

**DOI:** 10.3389/fmed.2021.709884

**Published:** 2021-08-02

**Authors:** Liyuan Long, Hai Li, Guohong Deng, Xianbo Wang, Sihong Lu, Beiling Li, Zhongji Meng, Yanhang Gao, Zhiping Qian, Feng Liu, Xiaobo Lu, Haotang Ren, Jia Shang, Hai Li, Shaoyang Wang, Yubao Zheng, Huadong Yan, Shan Yin, Wenting Tan, Qun Zhang, Xin Zheng, Jinjun Chen, Sen Luo, Jinming Zhao, Wei Yuan, Tao Li, Rongjiong Zheng, Junping Liu, Xiaoxiao Liu, Wenyi Gu, Sumeng Li, Xue Mei, Ruochan Chen, Yan Huang

**Affiliations:** ^1^Department of Infectious Diseases, Hunan Key Laboratory of Viral Hepatitis, Xiangya Hospital, Central South University, Changsha, China; ^2^Department of Gastroenterology, School of Medicine, Ren Ji Hospital, Shanghai Jiao Tong University, Shanghai, China; ^3^Key Laboratory of Gastroenterology and Hepatology, Shanghai Institute of Digestive Disease, Chinese Ministry of Health (Shanghai Jiao Tong University), Shanghai, China; ^4^Chinese Chronic Liver Failure (CLIF) Consortium, Shanghai, China; ^5^Department of Infectious Diseases, Southwest Hospital, Third Military Medical University (Army Medical University), Chongqing, China; ^6^Center of Integrative Medicine, Beijing Ditan Hospital, Capital Medical University, Beijing, China; ^7^Department of Infectious Diseases, Institute of Infection and Immunology, Tongji Medical College, Union Hospital, Huazhong University of Science and Technology, Wuhan, China; ^8^Hepatology Unit, Department of Infectious Diseases, Nanfang Hospital, Southern Medical University, Guangzhou, China; ^9^Department of Infectious Diseases, Taihe Hospital, Hubei University of Medicine, Shiyan, China; ^10^Department of Hepatology, The First Hospital of Jilin University, Changchun, China; ^11^Department of Liver Intensive Care Unit, Shanghai Public Health Clinical Centre, Fudan University, Shanghai, China; ^12^Department of Hepatology, Tianjin Institute of Hepatology, Nankai University Second People's Hospital, Tianjin, China; ^13^Department of Infectious Diseases and Hepatology, The Second Hospital of Shandong University, Jinan, China; ^14^Infectious Disease Center, The First Affiliated Hospital of Xinjiang Medical University, Ürümqi, China; ^15^State Key Laboratory for Diagnosis and Treatment of Infectious Diseases, Collaborative Innovation Center for Diagnosis and Treatment of Infectious Disease, The First Affiliated Hospital, Zhejiang University School of Medicine, Hangzhou, China; ^16^Department of Infectious Diseases, Henan Provincial People's Hospital, Zhengzhou, China; ^17^Department of Infectious Diseases, Affiliated Hospital of Logistics University of People's Armed Police Force, Tianjin, China; ^18^Department of Infectious Diseases, Fuzhou General Hospital of Nanjing Military Command, Fuzhou, China; ^19^Department of Infectious Diseases, The Third Affiliated Hospital, Sun Yat-sen University, Guangzhou, China; ^20^Department of Hepatology, Ningbo No. 2 Hospital, Ningbo, China

**Keywords:** hepatic encephalopathy, brain failure, acute on chronic liver disease, prospective, multicenter

## Abstract

**Importance:** Hepatic encephalopathy is a severe complication, and its contribution to clinical adverse outcomes in patients with acute-on-chronic liver diseases from the East is unclear.

**Objective:** We aimed to investigate the impact of hepatic encephalopathy on clinical characteristics and adverse outcomes in prospective and multicenter cohorts of patients with acute-on-chronic liver diseases.

**Design:** We conducted a cohort study of two multicenter prospective cohorts.

**Setting:** China.

**Participants:** Acute-on-chronic liver disease patients with various etiologies.

**Exposure:** The diagnosis and severity of hepatic encephalopathy were assessed using the West Haven scale.

**Main Outcome Measure:** The correlation between clinical adverse outcomes and varying hepatic encephalopathy grades was analyzed in the target patients.

**Results:** A total of 3,949 patients were included, and 340 of them had hepatic encephalopathy. The incidence of hepatic encephalopathy was higher in patients with alcohol consumption (9.90%) than in those with hepatitis B virus infection (6.17%). The incidence of 28- and 90-day adverse outcomes increased progressively from hepatic encephalopathy grades 1–4. Logistic regression analysis revealed that hepatic encephalopathy grades 3 and 4 were independent risk factors for the 28- and 90-day adverse outcome in the fully adjusted model IV. Stratified analyses showed similar results in the different subgroups. Compared to grades 1–2 and patients without hepatic encephalopathy, those with grade 3 hepatic encephalopathy had a significant increase in clinical adverse outcomes, independent of other organ failures.

**Conclusions and Relevance:** Hepatic encephalopathy grades 3–4 were independent risk factors for 28- and 90-day adverse outcomes. Hepatic encephalopathy grade 3 could be used as an indicator of brain failure in patients with acute-on-chronic liver disease.

## Key Points

- **Question:** What is the contribution of hepatic encephalopathy grades to clinical adverse outcomes in patients with acute-on-chronic liver diseases from the East.
- **Findings:** Logistic regression analysis revealed that hepatic encephalopathy grades 3 and 4 were independent risk factors for the 28- and 90-day adverse outcome of patients with acute-on-chronic liver diseases in the fully adjusted model IV.
- **Meaning:** Prevention of progression to higher grades of HE should be an important therapeutic goal. Hepatic encephalopathy grade 3 could be used as an indicator of brain failure in patients with acute-on-chronic liver disease.

## Introduction

Chronic liver disease (CLD) is currently an increasing global problem and burden (1–3). Acute event is a common clinical condition in patients with CLDs, and subsequently progresses to severe liver injury or even liver failure if the condition continues to be aggravated (4). Patients with CLD and acute events are considered to have acute-on-chronic liver diseases (AoCLD) (5). Hepatic encephalopathy (HE) remains one of the most complex and worrisome complications due to severe hepatocellular dysfunction, the presence of large portal-systemic shunts, or both. It usually presents with a wide spectrum of neurological/psychiatric abnormalities, ranging from subclinical alterations, sleep disturbances, personality changes, abnormal behaviors, and coma (6, 7). A combined clinical practice guideline of the European Association for the Study of the Liver (EASL) and American Association for the Study of Liver Disease concluded that HE prevalence could increase as high as 80% in the course of follow-up, whereas overt HE will occur in 30–40% of patients with cirrhosis during their overall clinical courses (8). HE leads to considerable mortality and exerts a multidimensional burden on patients, their caregivers, and the national healthcare system (9, 10).

Considering the recent advances in treatment available for HE, the higher proportion of cognitively impaired older patients with AoCLD, and the increased significance of HE with the relative reduction in variceal bleeding, re-evaluation of HE-associated prognosis is necessary (9). The contribution of HE to clinical outcome as an independent risk factor is important for analysis in a multicenter eastern setting because the etiologies of CLDs, management strategies, and available therapeutic options for HE in the eastern region differ from those around the world (4). To define the impact of HE on characteristics and adverse outcomes, we evaluated two multicenter prospective cohorts including 3,970 patients with CLD (both cirrhotic and non-cirrhotic) with various etiologies and acute events in China.

## Methods

### Study Design and Patients

Patients were recruited from two prospective multicenter cohorts with acute events of CLD, named CATCH-LIFE (NCT02457637 and NCT03641872), established by the Chinese Chronic Liver Failure Consortium composed of 15 tertiary hospitals in hepatitis B virus (HBV) endemic areas from January 2015 to December 2017 and July 2018 to January 2019, respectively (11, 12). The study was approved by the Renji Hospital Ethics Committee of Shanghai Jiaotong University School of Medicine [ethics codes: (2014)148k and (2016)142k], and all written consent was obtained from the patients.

**Figure 1:**
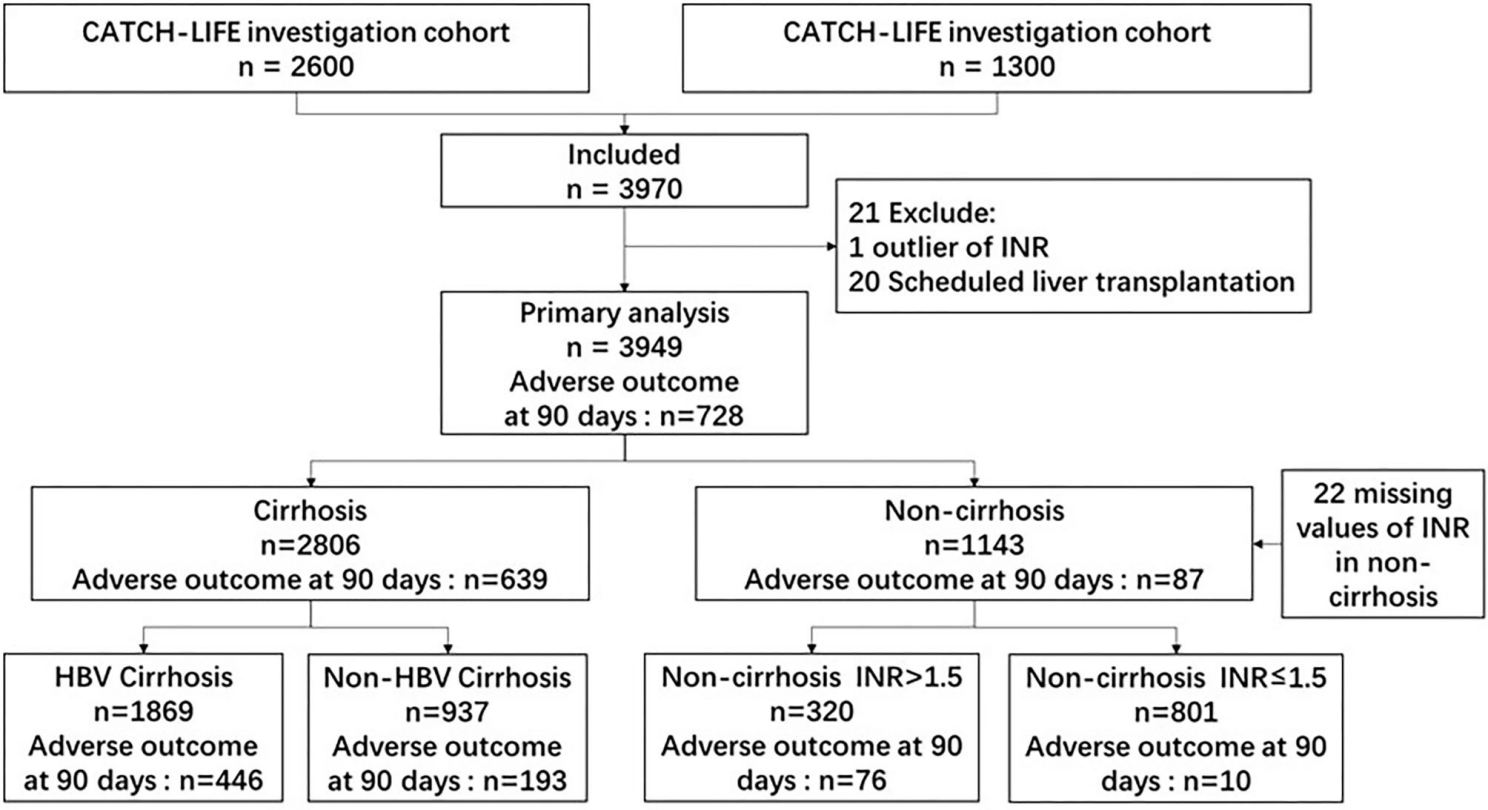
Flow chart of patient recruitment. INR, international normalized ratio; HBV, hepatitis B virus.

Together, the two independent cohort studies enrolled 3,970 patients with acute events of CLD, with 2,600 and 1,370 patients in the first and second cohorts, respectively. Acute events of CLD were defined as CLD with acute decompensation (AD) or acute liver injury (ALI). CLD was defined as cirrhotic or non-cirrhotic liver disease with a history of liver dysfunction lasting >6 months. Cirrhosis was diagnosed based on computed tomography/magnetic resonance imaging findings, laboratory test results, clinical symptoms, and history of liver disease. A diagnosis of CATCH-LIFE-defined AD required individuals to have acute development of gastrointestinal (GI) hemorrhage, HE, ascites, infection (e.g., spontaneous peritonitis and pneumonia), jaundice [total bilirubin (TB) level > 5 mg/dL], or any combination of these within 1 month before enrollment (13, 14). ALI was defined when total bilirubin was >2 mg/dl or alanine aminotransferase/aspartate aminotransferase was 3 times the range within 1 week (12, 15). The exclusion criteria were hepatocellular carcinoma or other liver malignancies before or during admission, extrahepatic malignancies or severe chronic extrahepatic disease, patients younger than 18 years of age or older than 80 years of age, and pregnancy. Among the 3,970 patients, we excluded one patient with an outlier of the international normalized ratio (INR) and 20 scheduled liver transplantations; thus, the final number of patients analyzed was 3,949. A flow chart of patient recruitment is shown in Figure 1.

### Data Collection and Diagnosis of HE

We collected clinical data from all enrolled patients, including clinical manifestations and laboratory measurements. HE was diagnosed based on impaired cognition, consciousness, or motor function. The diagnosis and severity of HE were assessed according to the West Haven scale (16, 17) and grouped into four levels (from grades I to IV). The day of admission was day 1. Clinical adverse outcomes were defined as patient mortality or liver transplantation.

### Statistical Analysis

Continuous variables were analyzed for normality using the Kolmogorov–Smirnov test. Normally distributed variables were compared using the Student *t*-test and are represented as the mean ± standard deviation. Non-normally distributed variables were compared using the Mann–Whitney U test and are presented as median with interquartile range (IQR). Categorical variables were compared using the χ^2^ or Fisher exact test and are represented as count and percentage. Univariate logistic regression analysis was used to determine the potential correlation between HE and 28- and 90-day adverse outcomes. Stepwise regression was used to build logistic regression model I-IV. Model I was unadjusted. Model II was adjusted for age, sex, and etiology. Model III was adjusted on the basis of model II plus cirrhosis, ascites, infection, and gastrointestinal bleeding. Model IV is adjusted based on model III plus total bilirubin, international normalized ratio, and creatinine. Every Model is adjusted stepwise to further demonstrate the influence of these parameters on adverse outcome. Model IV is adjusted based on model III plus the parameters with clinical significance as indicated in other study, like TB, INR and Cr (18). The survival rates in patients with different HE grades were estimated using the Kaplan–Meier method. All statistical analyses were performed with R (version 4.0.2, http://www.r-project.org).

## Results

### Clinical Characteristics and Adverse Outcome of Patients With Acute-on-Chronic Liver Diseases According to the HE Grades

Among 3,949 patients with AoCLD, including those with and without cirrhosis, 340 developed HE. The incidences of HE grades 1, 2, 3, and 4 were 37.35, 40.29, 16.18, and 5.88%, respectively. People with HE grades 1–4 were older than those without HE (*p* < 0.001), and male patients were more common than female patients in HE grades 1–4. Regarding etiology, the top three were HBV infection, alcohol consumption, and autoimmune disease. Among patients with HE grades 1–4, there was a high incidence of AD, such as infection and jaundice (Table 1).

**Table 1:** Association of HE grade with characteristics of AoCLD patients.

**Characteristics**	**NO HE (*N* = 3,610)**	**HE 1 (*N* = 127)**	**HE 2 (*N* = 137)**	**HE 3 (*N* = 55)**	**HE 4 (*N* = 20)**
**Demographic**					
Age	48.00 (39.81, 57.25)	53.01 (45.18, 61.08)	52.51 (44.08, 60.00)	49.74 (40.45, 61.91)	51.16 (42.20, 55.66)
Gender (male)	2,638 (73.1)	101 (79.5)	112 (81.8)	44 (80.0)	15 (75.0)
**Etiology**					
HBV	2,600 (72.0)	74 (58.3)	87 (63.5)	26 (47.3)	11 (55.0)
Alcohol	628 (17.4)	35 (27.6)	38 (27.7)	19 (34.5)	4 (20.0)
Autoimmune	363 (10.1)	10 (7.9)	8 (5.8)	7 (12.7)	3 (15.0)
HCV	129 (3.6)	7 (5.5)	6 (4.4)	1 (1.8)	0 (0.0)
HEV	79 (2.2)	3 (2.4)	5 (3.6)	0 (0.0)	1 (5.0)
NAFLD	146 (4.0)	4 (3.1)	0 (0.0)	1 (1.8)	0 (0.0)
Schistosomiasis	48 (1.3)	4 (3.1)	2 (1.5)	1 (1.8)	0 (0.0)
Cryptogenic	171 (4.7)	9 (7.1)	9 (6.6)	5 (9.1)	2 (10.0)
**Cirrhosis status**					
Non_cirrhosis	1,101 (30.5)	17 (13.4)	13 (9.5)	8 (14.5)	4 (20.0)
Cirrhosis	2,509 (69.5)	110 (86.6)	124 (90.5)	47 (85.5)	16 (80.0)
Compensated	224 (6.2)	0 (0.0)	0 (0.0)	0 (0.0)	0 (0.0)
Decompensated	2,285 (63.3)	110 (86.6)	124 (90.5)	47 (85.5)	16 (80.0)
**Acute decompensation**					
Infection	740 (20.5)	29 (22.8)	49 (35.8)	17 (30.9)	6 (30.0)
Jaundice	1,662 (46.1)	57 (44.9)	85 (62.0)	39 (70.9)	13 (65.0)
Ascites	1,692 (46.9)	57 (44.9)	67 (48.9)	24 (43.6)	10 (50.0)
GI_Bleeding	530 (14.7)	16 (12.6)	20 (14.6)	9 (16.4)	3 (15.0)
**Laboratory tests**					
TB	4.13 (1.52, 13.38)	3.71 (2.18, 16.02)	10.36 (2.40, 26.21)	10.07 (3.86, 26.52)	13.11 (3.44, 23.41)
INR	1.40 (1.17, 1.76)	1.58 (1.41, 2.22)	1.84 (1.40, 2.79)	1.98 (1.48, 2.98)	2.13 (1.75, 3.01)
CR	0.77 (0.64, 0.93)	0.78 (0.62, 0.96)	0.84 (0.66, 1.11)	0.88 (0.64, 1.32)	0.78 (0.66, 1.08)
BUN	4.49 (3.40, 6.23)	5.05 (3.53, 8.02)	5.99 (4.25, 8.26)	7.11 (3.82, 15.09)	7.36 (4.55, 10.74)
ALB	32.30 (28.20, 37.00)	31.00 (27.00, 34.60)	30.20 (26.80, 34.30)	28.30 (26.70, 32.10)	31.70 (26.45, 35.39)
ALT	100.3 (34.4, 436.0)	45.2 (23.0, 147.0)	45.0 (25.7, 140.6)	57.0 (26.2, 152.2)	102.3 (39.0, 399.3)
AST	113.0 (49.0, 292.0)	75.9 (35.3, 181.1)	76.5 (38.4, 159.8)	89.0 (46.4, 181.2)	133.9 (70.0, 251.5)
WBC	4.95 (3.60, 6.86)	4.91 (3.60, 6.91)	6.36 (4.09, 9.99)	6.90 (5.19, 12.01)	9.44 (6.36, 13.07)
PLT	96.0 (59.0, 148.0)	78.0 (50.4, 118.5)	69.0 (48.0, 119.0)	79.0 (56.5, 128.5)	84.5 (43.6, 112.2)
FIO_2_	476.2 (466.7, 476.2)	471.4 (461.9,476.2)	476.2 (466.7, 476.2)	471.4 (433.3, 476.2)	466.7 (410.3, 476.2)
MAP	89.00 (83.00, 94.67)	89.00 (80.66, 96.67)	87.00 (82.00, 94.00)	90.00 (76.84, 96.34)	94.50 (87.83, 101.75)
AKP	127.0 (91.0, 172.0)	116.0 (94.0, 163.4)	121.7 (83.7, 162.1)	117.2 (83.4, 161.2)	113.0 (80.0, 158.5)
γ-GT	82.00 (40.05, 155.88)	60.00 (25.50, 115.00)	48.00 (25.23, 93.40)	44.00 (30.60, 92.60)	54.90 (33.50, 69.50)
NL_ratio	2.46 (1.54, 4.24)	3.03 (1.81, 5.43)	4.33 (2.64, 7.74)	5.61 (3.23, 7.83)	9.03 (4.43, 11.72)
Hemoglobin	118.0 (97.0, 136.0)	113.0 (92.5, 128.5)	108.0 (84.0, 129.0)	98.0 (78.6, 120.5)	109.7 (97.2, 130.7)
Neutrophil	2.99 (1.99, 4.55)	3.10 (2.17, 5.12)	4.57 (2.46, 7.76)	5.39 (3.15, 9.03)	7.18 (4.25, 10.88)
Lymphocyte	1.22 (0.81, 1.75)	1.09 (0.76, 1.46)	1.00 (0.68, 1.50)	1.19 (0.70, 1.83)	0.96 (0.71, 1.12)
K	3.87 (3.52, 4.20)	3.81 (3.50, 4.12)	3.82 (3.41, 4.28)	4.00 (3.70, 4.39)	3.75 (3.14, 4.16)
Na	138.3 (135.7, 141.0)	137.9 (135.0,140.9)	136.0 (133.0, 141.0)	135.2 (131.3, 139.0)	138.7 (135.0, 141.5)
**Scores**					
MELD	15.50 (10.00, 22.00)	16.00 (12.00, 27.00)	21.50 (13.75, 30.25)	24.50 (18.00, 33.75)	23.00 (17.00, 31.00)
MELD_Na	17.00 (11.00, 24.00)	19.00 (13.00, 29.00)	24.00 (15.00, 32.00)	27.00 (16.00, 35.00)	22.00 (16.00, 33.00)
IMELD	34.00 (27.00, 41.00)	37.00 (30.00, 45.00)	41.00 (34.00, 50.50)	47.00 (35.00, 56.00)	42.00 (34.50, 47.50)
CLIF_SOFA	5.00 (3.00, 6.00)	6.00 (5.00, 8.00)	9.00 (6.75, 9.00)	10.00 (8.00, 11.75)	11.00 (9.00, 12.00)
SOFA	8.00 (7.00, 9.00)	9.00 (8.00, 10.00)	10.00 (9.00, 11.00)	10.00 (9.00, 11.00)	9.00 (8.00, 11.50)
CHILD_PUGH	8.00 (7.00, 10.00)	10.00 (8.00, 11.75)	11.00 (9.00, 12.00)	12.00 (11.00, 13.00)	11.50 (11.00, 13.00)

Patients with and without HE showed significant differences in several laboratory variables. Compared to the patients without HE, those with HE grades 1–4 showed a gradual increase in leukocyte counts, more deteriorated liver parameters (bilirubin, albumin, and INR), and progressively decreased hemoglobin and platelet (PLT) values. Renal function was also significantly worse in patients with HE grades 1–4 than in those without HE (*p* < 0.001). Other alterations in parameters indicated a statistically significant frequency of organ failure and unstable internal environment, such as respiratory failure, renal function, and sodium ion concentration, in patients with HE grades 1–4 compared to those without HE. At inclusion, liver function assessed with the Model of End-stage Liver Disease (MELD), Chronic Liver Failure (CLIF)-Sequential Organ Failure Assessment (SOFA), SOFA score, and Child-Pugh score were significantly worse among the HE groups than among the non-HE group, especially in HE grades 1–4 (Table 1).

**Figure 2:**
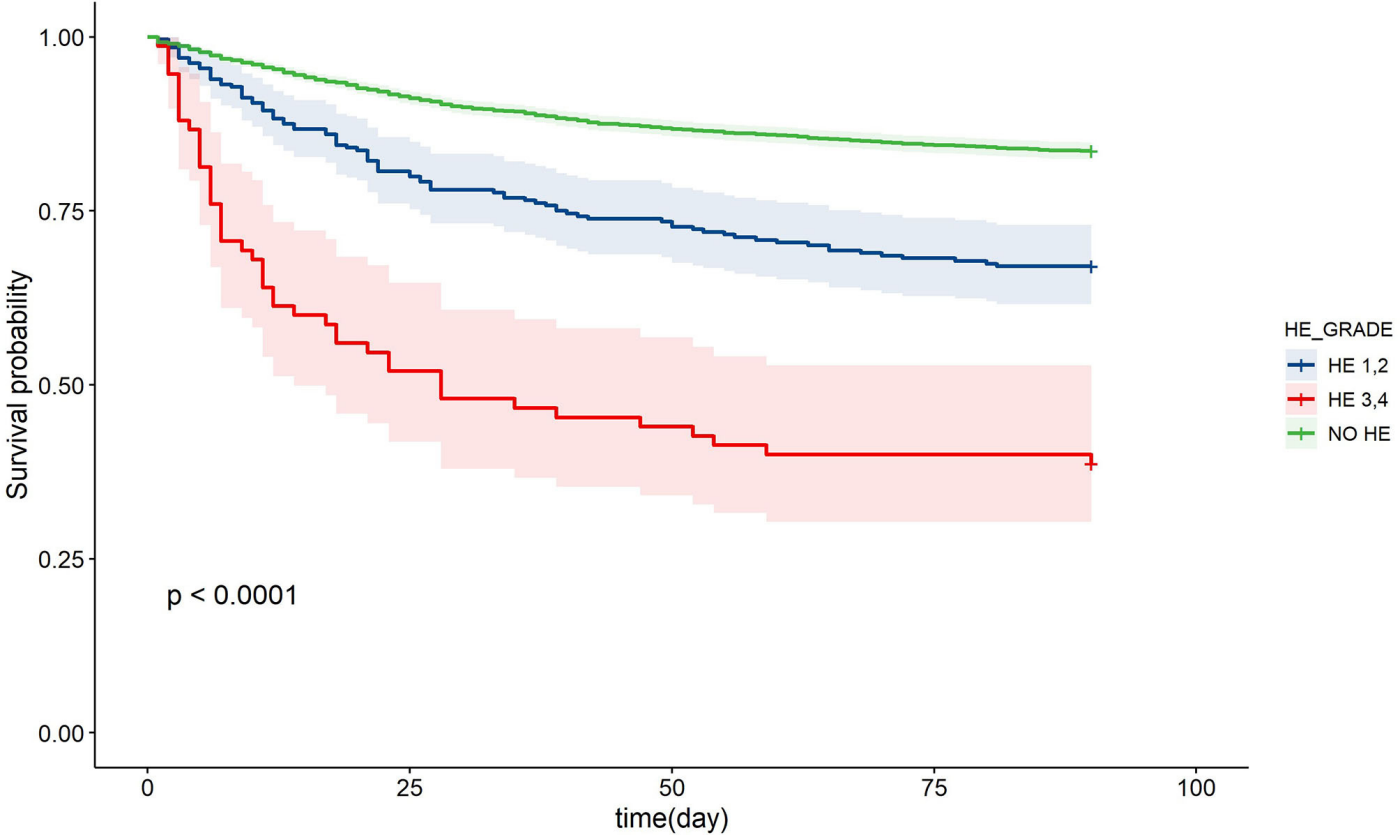
Kaplan-Meier curve of patients with chronic liver disease and acute events in different HE grades. HE, hepatic encephalopathy.

Next, we sought to assess the association of HE grades with 28- and 90-day adverse outcomes in patients with AoCLD. The incidence of 28- and 90-day adverse outcomes increased progressively from HE grades 1 to 4 (Table 2). The Kaplan–Meier curve also revealed that the survival rate was negatively correlated with HE grades, indicating a poorer prognosis in the HE group than in the non-HE group (Figure 2).

**Table 2:** Association of HE grade with 28-day outcome and 90-day outcome of AoCLD patients.

**Outcome**	**NO HE (*N* = 3,610)**	**HE1 (*N* = 127)**	**HE2 (*N* = 137)**	**HE3 (*N* = 55)**	**HE4 (*N* = 20)**
28-day adverse outcome	351 (9.7)	23 (18.1)	35 (25.5)	26 (47.3)	13 (65.0)
28-day LT	147 (4.1)	5 (3.9)	4 (2.9)	7 (12.7)	2 (10.0)
28-day die	204 (5.7)	18 (14.2)	31 (22.6)	19 (34.5)	11 (55.0)
90-day adverse outcome	594 (16.5)	36 (28.3)	52 (38.0)	32 (58.2)	14 (70.0)
90-day LT	204 (5.7)	7 (5.5)	6 (4.4)	7 (12.7)	2 (10.0)
90-day die	390 (10.8)	29 (22.8)	46 (33.6)	25 (45.5)	12 (60.0)

The major etiologies contributing to HE are different in the Eastern and Western hemispheric regions. We further investigated the impact of HBV infection and alcohol consumption, the two major etiologies of CLD, on the clinical features of patients with HE. The incidence of HE was higher in patients with alcohol consumption (9.90%) than in patients with HBV infection (6.17%). Patients with alcohol-associated HE were older than those with HBV-associated HE (*p* = 0.008), and male patients were more common than female patients in the alcohol-associated HE group (*p* = 0.004). Jaundice was more common in patients with HBV-associated HE than in those with alcohol-associated HE (*p* = 0.017), while GI bleeding was more common in patients with alcohol-associated HE than in those with HBV-associated HE (*p* = 0.001). The values of TB, INR, alanine aminotransferase (ALT), aspartate aminotransferase (AST), MELD, MELD and sodium (MELD-Na), and integrated MELD (IMELD) were significantly higher in the HBV-associated HE group than in the alcohol-associated HE group (*p* < 0.05), suggesting worse clinical conditions with HBV-associated HE. The value of gamma-glutamyl transferase was higher, whereas that of hemoglobin was lower in patients with alcohol-associated HE than in those with HBV-associated HE. Moreover, the incidence of 28- and 90-day adverse outcomes was higher in patients with HBV-related HE than in those with alcohol-related HE (*p* = 0.003 and *p* = 0.041, respectively) (Table 3).

**Table 3:** Characteristics of HE patients with different etiologies.

	**HE**			**HE**		
**Characteristics**	**Alcohol (*n* = 69)**	**HBV (*n* = 171)**	***P*-value**	**Non-cirrhosis (*n* = 42)**	**Cirrhosis (*n* = 297)**	***P*-value**
**Demographic**						
Age	54.3 (48.0, 59.0)	50.5 (39.7, 56.8)	0.008	39.5 (33.5, 46.9)	53.3 (46.7, 61.1)	<0.001
Gender (male)	68 (98.6)	144 (84.2)	0.004	32 (76.2)	240 (80.8)	0.62
**HE_GRADE**			0.319			0.479
HE 1	23 (33.3)	62 (36.3)		17 (40.5)	110 (37.0)	
HE 2	27 (39.1)	76 (44.4)		13 (31.0)	124 (41.8)	
HE 3	16 (23.2)	23 (13.5)		8 (19.0)	47 (15.8)	
HE 4	3 (4.3)	10 (5.8)		4 (9.5)	16 (5.4)	
**AD**						
Infection	19 (27.5)	50 (29.2)	0.915	13 (31.0)	88 (29.6)	1
Jaundice	33 (47.8)	112 (65.5)	0.017	38 (90.5)	156 (52.5)	<0.001
Ascites	37 (53.6)	78 (45.6)	0.326	12 (28.6)	146 (49.2)	0.019
GI-Bleeding	19 (27.5)	17 (9.9)	0.001	0 (0.0)	48 (16.2)	0.01
**Laboratory tests**						
TB	4.77 (2.16, 10.36)	14.09 (2.83, 26.68)	<0.001	21.96 (14.61, 28.94)	5.49 (2.18, 22.58)	<0.001
INR	1.61 (1.35, 2.09)	2.14 (1.52, 2.91)	<0.001	2.62 (1.87, 3.19)	1.70 (1.40, 2.41)	<0.001
CR	0.81 (0.65, 1.05)	0.81 (0.63, 1.04)	0.63	0.87 (0.67, 1.00)	0.79 (0.64, 1.10)	0.518
BUN	6.31 (4.60, 11.76)	5.21 (3.80, 7.67)	0.012	3.90 (2.47, 6.38)	5.94 (4.13, 10.20)	<0.001
ALB	30.0 (27.40, 33.00)	30.8 (27.00, 34.75)	0.554	32.15 (29.10, 35.66)	29.90 (26.60, 33.50)	0.005
ALT	30.0 (18.00, 58.00)	81.0 (27.30, 426.35)	<0.001	287.5 (106.7, 1,183.8)	42.0 (23.0, 111.7)	<0.001
AST	51.6 (32.00, 102.22)	101.1 (48.50, 239.85)	<0.001	187.9 (122.3, 535.3)	65.0 (35.6, 146.0)	<0.001
WBC	5.98 (4.30, 8.90)	6.10 (3.77, 10.09)	0.695	8.81 (5.63, 11.22)	5.89 (3.76, 9.20)	0.001
PLT	68.0 (46.40, 99.40)	82.0 (51.20, 121.00)	0.18	121.0 (91.8, 190.0)	71.0 (45.4, 112.0)	<0.001
FIO_2	471.4 (457.1, 476.2)	471.4 (461.9, 476.2)	0.362	476.2 (466.7, 476.2)	471.4 (461.9, 476.2)	0.407
MAP	91.0 (76.67, 96.00)	89.0 (82.66, 96.84)	0.219	89.0 (83.0, 99.3)	89.0 (81.0, 96.0)	0.361
AKP	105.0 (74.5,152.7)	125.5 (98.0, 162.7)	0.024	134.5 (97.7, 168.1)	117.0 (83.0, 158.0)	0.159
γ-GT	86.8 (34.4, 190.3)	46.2 (26.3, 81.9)	<0.001	68.9 (43.8, 91.6)	48.6 (24.0, 99.0)	0.035
NL_ratio	5.13 (3.11, 7.62)	3.82 (2.26, 7.03)	0.054	4.62 (2.83, 7.71)	3.90 (2.31, 7.10)	0.176
Hemoglobin	88.0 (76.2, 115.2)	116.0 (98.0, 135.0)	<0.001	128.0 (105.8, 145.3)	106.0 (84.0, 125.0)	<0.001
Neutrophil	4.20 (2.96, 7.08)	4.23 (2.24, 8.00)	0.481	6.16 (3.88, 8.93)	3.87 (2.27, 6.36)	0.001
Lymphocyte	0.86 (0.71, 1.31)	1.10 (0.73, 1.52)	0.05	1.27 (0.97, 1.61)	1.00 (0.69, 1.45)	0.043
K	3.87 (3.48, 4.34)	3.88 (3.58, 4.27)	0.987	3.82 (3.63, 4.17)	3.83 (3.46, 4.24)	0.777
Na	136.7 (132.9, 140.5)	137.0 (133.2, 141.0)	0.702	137.8 (136.1, 140.9)	136.9 (133.0, 141.0)	0.06
**Scores**						
MELD	15.0 (8.8, 19.3)	25.5 (16.0, 31.0)	<0.001	29.0 (24.0, 32.0)	19.0 (13.0, 29.0)	<0.001
MELD_Na	16.0 (10.0, 23.5)	27.0 (16.0, 33.0)	<0.001	30.0 (27.0, 33.0)	21.00 (13.5, 29.0)	<0.001
IMELD	36.0 (27.0, 41.5)	43.0 (35.5, 51.0)	<0.001	43.0 (38.0, 50.0)	39.0 (33.0, 50.0)	0.049
CLIF_SOFA	7.0 (6.0, 9.3)	9.0 (7.0, 10.0)	0.09	9.0 (8.0, 11.0)	8.0 (6.0, 9.0)	0.003
SOFA	10.0 (8.0, 11.0)	10.0 (9.0, 11.0)	0.165	10.0 (9.0, 11.0)	10.0 (9.0, 11.0)	0.846
CHILD_PUGH	11.0 (9.0, 12.0)	11.0 (9.0, 12.0)	0.249	11.0 (10.0, 13.0)	11.0 (9.0, 12.0)	0.117
**Outcome**						
O28	9 (13.0)	56 (32.7)	0.003	18 (42.9)	79 (26.6)	0.046
O90	19 (27.5)	73 (42.7)	0.041	20 (47.6)	114 (38.4)	0.328

The incidence of HE was higher in cirrhotic patients than in non-cirrhotic patients (10.58 vs. 3.76%). Patients with cirrhosis-associated HE were older than patients with non-cirrhosis-associated HE (*p* < 0.001). Jaundice, ascites, and GI bleeding were more common in patients with cirrhosis-associated HE than in those with non-cirrhosis-associated (*p* < 0.05). The values of most laboratory tests, such as TB, INR, blood urea nitrogen, ALT, AST, white blood cell, and PLT, and clinical scores such as MELD, MELD-Na, IMELD, and CLIF-SOFA were worse in cirrhotic patients than in non-cirrhotic patients, as expected. There was no obvious difference in the incidence of 28- and 90-day adverse outcomes between patients with and without cirrhosis (Table 3).

### Role of HE as an Independent Risk Factor of Adverse Outcome in Patients With Acute-on-Chronic Liver Diseases

**Figure 3:**
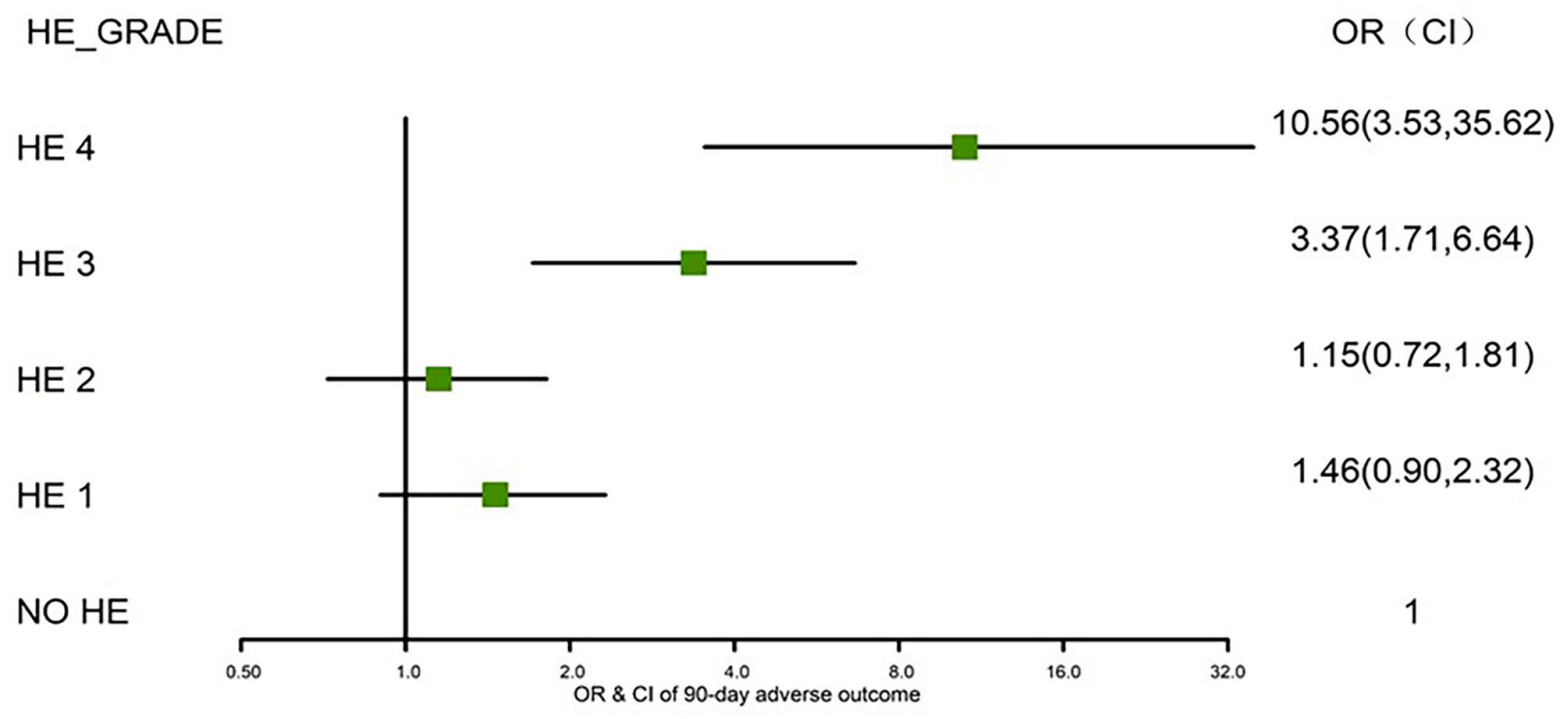
Risks of 90-day adverse outcomes by HE grades I–IV (fully adjusted). HE, hepatic encephalopathy; OR, odds ratio; CI, confidence interval.

We further assessed the role of HE as an independent risk factor for adverse outcomes in patients with AoCLD. We gradually controlled for other risk factors such as age, sex, etiology, cirrhosis, ascites, infection, GI bleeding, TB, INR, and creatinine (CR) in models I–IV. Univariate analysis using logistic regression revealed that HE grades 1–4 were all independent prognostic factors of 28-day adverse outcomes in models I–III (Table 4). Moreover, the odds ratio (OR) increased significantly as the HE grade progressed. In model IV, adjusted for model III plus values of TB, INR, and CR, HE grades 3 and 4 were significantly correlated with 90-day adverse outcomes, and the ORs were 4.03 (*p* < 0.0001) and 16.74 (*p* < 0.0001), respectively. HE grades 3 and 4 were also independent prognostic factors in the 90-day adverse outcome analysis in the fully adjusted model IV (Table 5 and Figure 3).

**Table 4:** Odds ratios and *p*-values of HE grades in the total population at day 28.

**HE_GRADE**	**Num of 28-day adverse outcome (%)**	**Model I**	**Model II**	**Model III**	**Model IV**
NO HE	351 (9.7)	1.0	1.0	1.0	1.0
1	23 (18.1)	2.05 (1.26, 3.20) 0.0024	1.99 (1.21, 3.13) 0.003	1.95 (1.18, 3.10) 0.005	1.47 (0.85, 2.46) 0.146
2	35 (25.5)	3.18 (2.11, 4.70) <0.0001	3.10 (2.04, 4.60) <0.0001	2.77 (1.82, 4.15) <0.0001	1.16 (0.68, 1.89) 0.563
3	26 (47.3)	8.32 (4.82, 14.30) <0.0001	8.76 (5.02, 15.21) <0.0001	8.80 (4.97, 15.54) <0.0001	4.03 (2.06, 7.78) <0.0001
4	13 (65.0)	17.24 (7.01, 46.15) <0.0001	18.76 (7.57, 50.58) <0.0001	19.04 (7.51, 52.44) <0.0001	16.74 (5.81, 52.31) <0.0001

*Model I, unadjusted*.

*Model II, adjusted for age, sex, and etiology*.

*Model III, adjusted for model II plus cirrhosis, ascites, infection, and gastrointestinal bleeding*.

*Model IV, adjusted for model III plus total bilirubin, international normalized ratio, and creatinine*.

**Table 5:** Odds ratios and *p*-values of HE grades in the total population at day 90.

**HE_GRADE**	**Num of 90-day adverse outcome (%)**	**Model I**	**Model II**	**Model III**	**Model IV**
NO HE	594 (16.5)	1.0	1.0	1.0	1.0
1	36 (28.3)	2.00 (1.34, 2.97) 0.0004	1.95 (1.29, 2.88) 0.003	1.95 (1.28, 2.91) 0.0014	1.46 (0.90, 2.32) 0.11
2	52 (38.0)	3.00 (2.10, 4.30) <0.0001	2.94 (2.04, 4.20) <0.0001	2.66 (1.82, 3.83) <0.0001	1.15 (0.72, 1.81) 0.54
3	32 (58.2)	7.10 (4.14, 12.34) <0.0001	7.26 (4.21, 12.73) <0.0001	7.65 (4.35, 13.56) <0.0001	3.37 (1.71, 6.64) 0.0004
4	14 (70.0)	11.90 (4.75, 33.70) <0.0001	12.66 (5.03, 35.99) <0.0001	13.07 (5.05, 38.10) <0.0001	10.56 (3.53, 35.62) <0.0001

*Model I, unadjusted*.

*Model II, adjusted for age, sex, and etiology*.

*Model III, adjusted for model II plus cirrhosis, ascites, infection, and gastrointestinal bleeding*.

*Model IV, adjusted for model III plus total bilirubin, international normalized ratio, and creatinine*.

**Figure 4:**
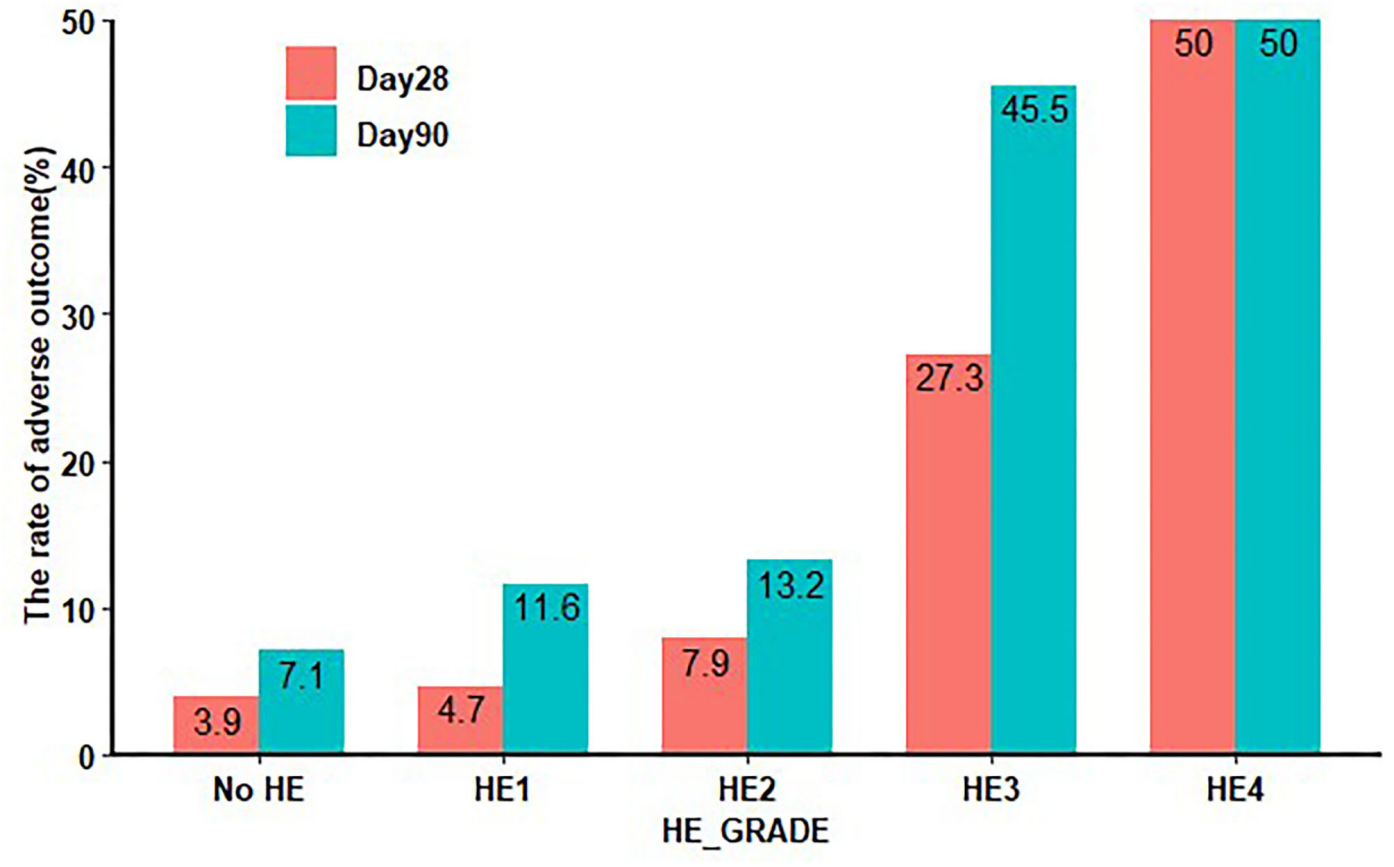
Twenty-eight-day and 90-day clinical adverse outcomes in HE excluding other organ failure. HE: TB level < 12 mg/dL, INR < 1.5, CR < 2 mg/dL, without respiratory failure. HE, hepatic encephalopathy; TB, total bilirubin; INR, international normalized ratio; CR, creatinine.

To investigate whether the contribution of HE to clinical adverse outcomes is independent of non-HE organ failures, such as hepatic, renal, pulmonary, and coagulation failures, we excluded patients with a TB level > 12 mg/dL, INR > 1.5, CR level > 2 mg/dL, and need for mechanical ventilation as respiratory failure, according to the definition of organ failure in prior studies (14, 19). We found that there was a significant increase in the clinical adverse outcomes in HE grades 3 and 4, when compared with grades 1–2 and patients without HE, both at 28 and 90 days (Figure 4).

### Stratified Analysis of 28- and 90-Day Adverse Outcomes by HE Grades

**Figure 5:**
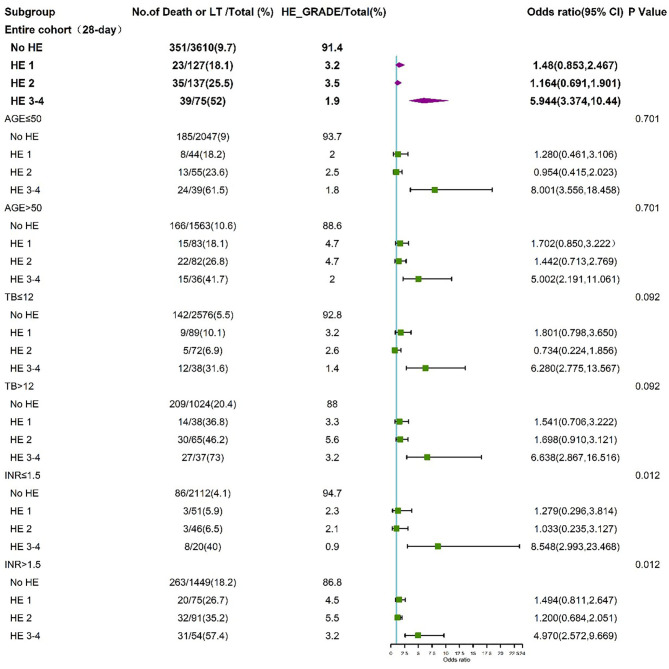
Stratified analyses of 28-day adverse outcomes by HE grades. HE, hepatic encephalopathy; TB, total bilirubin; INR, international normalized ratio; CI, confidence interval.

**Figure 6:**
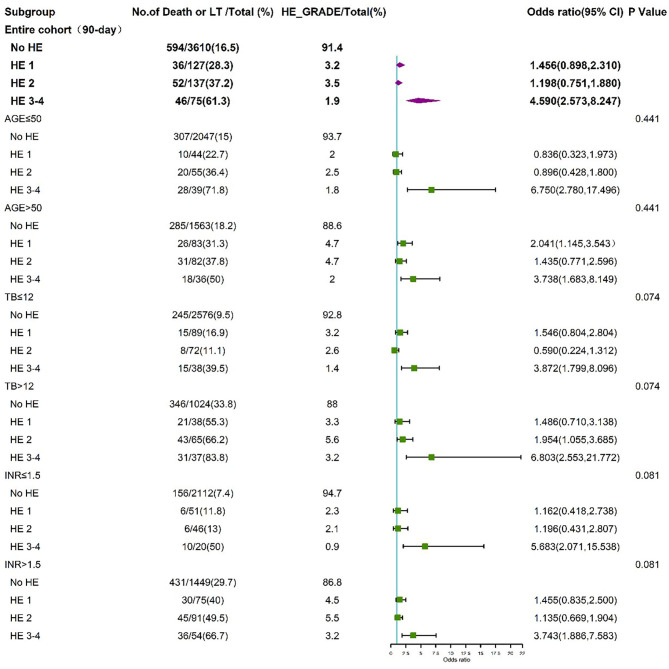
Stratified analyses of 90-day adverse outcome by HE grades. HE, hepatic encephalopathy; TB, total bilirubin; INR, international normalized ratio; CI, confidence interval.

To further assess the impact of HE grades on clinical adverse outcomes, we performed stratified analysis for interaction with HE grades for the individual related risk factors, including age (>50 and <50 years), TB level (>12 and <12 mg/dL), INR (>1.5 and <1.5), and HBV (HBV-related cirrhosis and non-HBV-related cirrhosis). The stratified analyses demonstrated that HE grades 3–4 but not HE grades 1–2 were independent risk factors for 28-day (Figure 5) and 90-day (Figure 6) adverse outcomes in all subgroups after fully adjusting for age, sex, etiology, cirrhosis, ascites, infection, GI bleeding, INR, and CR.

HBV was the main etiology (58.5%) in our cohort, and patients with cirrhosis constituted 71.1% of the entire group; thus, we further investigated the impact of HBV on the risk of HE in patients with cirrhosis. Multivariable logistic regression revealed that HE grades 3–4 were independent risk factors for 90-day adverse outcomes after full adjustment, both in patients with HBV-related (Table 6) and non-HBV-related cirrhosis (Table 7).

**Table 6:** Odds ratios and *p*-values of HE grades in patients with HBV-associated cirrhosis at day 90.

**HE_GRADE**	**Num of 90-day adverse outcome (%)**	**Model I**	**Model II**	**Model III**	**Model IV**
NO HE	375 (22.0)	1.0	1.0	1.0	1.0
1	19 (32.8)	1.72 (0.96, 2.98) 0.055	1.61 (0.90, 2.80) 0.094	1.70 (0.94, 2.98) 0.066	1.57 (0.79, 3.03) 0.177
2	30 (40.0)	2.36 (1.45, 3.79) 0.0003	2.34 (1.44, 3.76) 0.0004	2.34 (1.43, 3.79) 0.0005	1.00 (0.54, 1.83) 0.975
3-4	22 (73.3)	9.76 (4.48, 23.52) <0.0001	9.70 (4.44, 23.43) <0.0001	9.95 (4.52, 24.18) <0.0001	6.51 (2.42, 18.97) 0.0003

*Model I, unadjusted*.

*Model II, adjusted for age, sex, and etiology*.

*Model III, adjusted for model II plus ascites, infection, and gastrointestinal bleeding*.

*Model IV, adjusted for model III plus total bilirubin, international normalized ratio, and creatinine*.

**Table 7:** Odds ratios and *p*-values of HE grades in non-HBV-associated cirrhosis at day 90.

**HE_GRADE**	**Num of 90-day adverse outcome (%)**	**Model I**	**Model II**	**Model III**	**Model IV**
NO HE	151 (18.8)	1.0	1.0	1.0	1.0
1	15 (28.8)	1.75 (0.91, 3.21) 0.079	1.87 (0.97, 3.46) 0.051	2.02 (1.03, 3.78) 0.032	1.68 (0.78, 3.40) 0.161
2	12 (24.5)	1.40 (0.68, 2.67) 0.327	1.58 (0.76, 3.06) 0.190	1.67 (0.79, 3.29) 0.150	1.11 (0.47, 2.39) 0.788
3–4	15 (45.5)	3.59 (1.75, 7.29) 0.0003	4.12 (1.98, 8.49) 0.0001	4.54 (2.16, 9.46) <0.0001	3.50 (1.51, 7.90) 0.002

*Model I, unadjusted*.

*Model II, adjusted for age, sex, etiology*.

*Model III, adjusted for model II plus ascites, infection, and gastrointestinal bleeding*.

*Model IV, adjusted for model III plus total bilirubin, international normalized ratio, and creatinine*.

Non-cirrhotic patients were stratified into two groups according to the INR values. Non-cirrhotic patients with an INR < 1.5 were excluded because of the limited number of patients with 90-day adverse outcomes (only 10 patients). In non-cirrhotic patients with an INR > 1.5, HE grades 3–4 were also an independent risk factor for 90-day adverse outcomes after adjusting for confounders (Table 8).

**Table 8:** Odds ratios and *p*-values of HE grades in non-cirrhosis with INR > 1.5 at day 90.

**HE_GRADE**	**Num of 90-day adverse outcome (%)**	**Model I**	**Model II**	**Model III**	**Model IV**
NO HE	56 (19.6)	1.0	1.0	1.0	1.0
1	2 (16.7)	0.81 (0.12, 3.21) 0.798	0.99 (0.14, 4.11) 0.992	1.03 (0.14, 4.57) 0.963	0.59 (0.08, 2.75) 0.548
2	8 (66.7)	8.17 (2.48, 31.54) 0.0008	9.24 (2.68, 37.18) 0.0006	8.23 (2.26, 34.73) 0.001	2.04 (0.41, 10.85) 0.380
3–4	9 (81.8)	18.40 (4.58, 122.93) 0.0002	22.05 (5.08, 154.13) 0.0001	26.43 (5.95, 186.50) <0.0001	7.07 (1.32, 54.05) 0.030

*Model I, unadjusted*.

*Model II, adjusted for age, sex, etiology*.

*Model III, adjusted for model II plus ascites, infection, and gastrointestinal bleeding*.

*Model IV, adjusted for model III plus total bilirubin, international normalized ratio, and creatinine*.

## Discussion

The increasing burden of CLD with acute event worldwide should raise concerns regarding the prevention of morbidity and mortality in these patients (4). Our study's results shed light on the characteristics and impact of HE in patients with AoCLD from two large multicenter cohorts from an area highly endemic for HBV infection. Our study indicated that HE grades 3–4 are independent risk factors for 28- and 90-day adverse outcomes, and HE grade 3 could be used as an indicator of brain failure in patients with CLDs and acute events. To our knowledge, our study is currently the largest prospective cohort of patients with acute events of CLD in the East.

The incidence of HE in patients with AoCLD was 8.61%. However, the overall prevalence and cumulative incidence of HE are difficult to define, depending on symptom variability, the tools used for detection and scoring, and objective bias (7). Interestingly, the MELD, MELD-Na, IMELD, SOFA, and Child-Pugh scores showed a decrease in HE grade 4 compared to HE grade 3, indicating an increasing incidence of clinical adverse outcomes. The same results have been obtained in patients waiting for liver transplantation, revealing an independent role of HE in survival (20). The incidence of HE is also affected by different etiologies and cirrhosis status. The incidence of HE was higher in patients with alcohol consumption than in patients with HBV infection, although most other clinical indicators, MELD scores, and clinical outcomes are worse in HBV groups, suggesting that HE may be an indicator of organ failure independent of other organ failure in patients with alcohol consumption.

HE is a marker of decompensated disease and is a component of the Child-Pugh-Turcotte scoring system (21). The impact of HE on mortality among patients with cirrhosis, acute-on-chronic liver failure, and end-stage liver diseases awaiting liver transplantation has been explored in several studies (9, 22–27). In the Canonic Study by the EASL, HE appeared as an isolated syndrome or as part of acute-on-chronic liver failure, with different characteristics and high mortality. In these two large experiences of multicenter cohorts, we found that higher HE grades continue to carry a poor prognosis, as expected. The same conclusion has been drawn in subgroups such as HBV-related and non-HBV-related cirrhotic patients and non-cirrhotic patients with an INR > 1.5. Our results are consistent with those of other studies showing that HE grades are positively correlated with adverse clinical outcomes (15, 25, 28). Moreover, HE grades 3 and 4 [severe HE (20)] remained independent determinants of mortality in patients with AoCLD, independent of age, sex, etiology, ascites, infection, GI bleeding, TB, INR, and CR. Thus, we elucidated the clinical importance and prognostic significance of HE in patients with AoCLD, based on solid evidence-based proof instead of clinical experience or expert opinion.

Although there are several scoring systems for grading the severity of HE, the West Haven Criteria are most commonly used (29, 30). The potential subjectivity of HE grade identification using the West Haven Criteria has made accurate diagnosis of HE difficult in clinical settings, especially in isolating HE grade 0 from grade 1, or distinguishing grade 1 from grade 2. This is the reason HE grade 0 was not displayed in our analysis. However, the differentiation between HE grades 3–4 and 1–2 has good reliability and is routinely performed in clinical practice (31). We also chose the admission grade of HE to define HE severity, which avoided multiple grades due to individual subjectivity and reduced intra-observer variability, especially for general practitioners. However, the maximum HE grade and duration of HE in hospitalized patients significantly affect mortality and even survival rate after transplantation (9, 10, 16). Thus, it is of great importance to develop a more objective tool that will improve the accuracy and reproducibility of HE severity assessment, thereby increasing the prognostic value of HE grades in clinical settings.

Since HE can exist with and without other non-HE organ failures, we further assessed the impact of HE on clinical adverse outcomes independent of other organ failures. Common non-HE organ failures include liver failure, renal failure, coagulation dysfunction, and respiratory failure. First, we adjusted the values of TB, INR, and CR in a multivariable logistic regression model and found that HE grades 3 and 4 remained independent factors in the clinical adverse outcomes of patients with AoCLD. Moreover, we found that any grade of HE, but especially the higher grades, remained associated with high clinical adverse, irrespective of non-HE-associated organ failure. This becomes even more obvious because HE grade 3 significantly worsened the mortality prognosis regardless of non-HE-associated organ failure from 7.9 to 27.3% within 28 days. Summarizing all these results, our findings indicated that after excluding patients with other organ failure, HE grade 3 remained an indicator of brain failure in referred patients. A previous study also found that added non-HE-associated organ failures increased the mortality rate but did not affect the impact of HE severity on mortality in multivariable analysis (9). Therefore, clinicians, intensivists, and hospital medicine specialists view HE grade 3 as an important risk factor for death regardless of other non-HE-associated organ failures to accurately prognosticate the patients (32). In addition, it is important for clinicians to make every effort to prevent the development of HE or its worsening over time to affect outcomes. Prevention of progression to higher grades of HE should be an important therapeutic goal.

There are some limitations to our study. First, the severity of HE is a dynamic process that changes with progression of disease or initiation of treatment, and attention should be paid to the severity of HE at several time points in the future. Second, the grading of HE severity may vary between different medical centers due to subjective bias. Despite these limitations, we conclude that HE grades 3–4 are an important determinant of 28- and 90-day adverse outcomes in patients with AoCLD, independent of other organ failures, based on solid evidence-based proof. Counseling of patients and caregivers to seek medical attention before the development of grade 3–4 HE and prevention of in-hospital development of grades 3–4 HE should continue to be an important therapeutic goal.

## Data Availability Statement

The original contributions generated for the study are included in the article/supplementary material, further inquiries can be directed to the corresponding author/s.

## Ethics Statement

The studies involving human participants were reviewed and approved by Renji Hospital Ethics Committee of Shanghai Jiaotong University School of Medicine [ethics codes: (2014)148k and (2016)142k]. The patients/participants provided their written informed consent to participate in this study. Written informed consent was obtained from the individual(s) for the publication of any potentially identifiable images or data included in this article.

## Author Contributions

LL (data curation: equal; formal analysis: lead; investigation: equal; methodology: lead; writing-original draft: equal). HL (2nd author), GD, XW, SLu, ZM, YG, FL, XLu, HY, XZ, YZ, and JC (conceptualization: equal; data curation: equal; funding acquisition: equal; investigation: equal). BL, JS, SW, SY, WT, QZ, SLuo, JZ, WY, TL, RZ, XLiu, WG, SLi, XM, HR, HL (14th author), and JL (data curation: equal; investigation: equal). ZQ (conceptualization: equal; data curation: equal; investigation: equal). RC (conceptualization: equal; writing–original draft: lead; writing–review and editing: lead). YH (conceptualization: lead; data curation: lead; investigation: lead; supervision: lead; writing–review and editing: equal). All authors read and approve the version to be published.

## Conflict of Interest

The authors declare that the research was conducted in the absence of any commercial or financial relationships that could be construed as a potential conflict of interest. The handling editor declared a shared affiliation with several of the authors SLu, XZ, and SLi.

## Publisher's Note

All claims expressed in this article are solely those of the authors and do not necessarily represent those of their affiliated organizations, or those of the publisher, the editors and the reviewers. Any product that may be evaluated in this article, or claim that may be made by its manufacturer, is not guaranteed or endorsed by the publisher.
